# A New Intronic Variant in ECEL1 in Two Patients with Distal Arthrogryposis Type 5D

**DOI:** 10.3390/ijms22042106

**Published:** 2021-02-20

**Authors:** Viola Alesi, Francesca Sessini, Silvia Genovese, Giusy Calvieri, Ester Sallicandro, Laura Ciocca, Maura Mingoia, Antonio Novelli, Paolo Moi

**Affiliations:** 1 Laboratory of Medical Genetics, Translational Cytogenomics Research Unit, Bambino Gesù Children’s Hospital, IRCCS, 00165 Rome, Italy; silvia.genovese@opbg.net (S.G.); giusy.calvieri@opbg.net (G.C.); ester.sallicandro@opbg.net (E.S.); laura.ciocca@opbg.net (L.C.); antonio.novelli@opbg.net (A.N.); 2 Pediatric Clinic and Rare Diseases, Brotzu Hospital, 09134 Cagliari, Italy; francesca.sessini@aob.it; 3 Department of Medical Sciences and Public Health, University of Cagliari, 09124 Cagliari, Italy; mauramingoia@gmail.com (M.M.); paolo.moi@aob.it (P.M.)

**Keywords:** ECEL1, c.1507-9G>A, distal arthrogryposis, DA5D, 2q37.1

## Abstract

Distal Arthrogryposis type 5D (DA5D) is characterized by congenital contractures involving the distal joints, short stature, scoliosis, ptosis, astigmatism, and dysmorphic features. It is inherited in an autosomal recessive manner, and it is a result of homozygous or compound heterozygous variants in the ECEL1 gene. Here, we report two patients of Sardinian origin harboring a new intronic homozygous variant in ECEL1 (c.1507-9G>A), which was predicted to affect mRNA splicing by activating a cryptic acceptor site. The frequency of the variant is very low in the general human population, and its presence in our families can be attributed to a founder effect. This study provides an updated review of the known causative mutations of the ECEL1 gene, enriching the allelic spectrum to include the noncoding sequence.

## 1. Introduction

Arthrogryposis multiplex congenital (AMC) refers to the congenital joint contractures or movement restrictions involving multiple areas of the body. It occurs in between 1/3000 and 1/5000 live births [1] in association with several distinct disorders, generally as a consequence of fetal akinesia. In fact, the restriction of intrauterine movements and fetal joint mobility may lead to an excessive connective tissue deposition around the joints, resulting in arthrogryposis. Environmental and genetic factors have been reported, spanning from intrauterine space limitations or drug exposure to the fetal brain, and neuromuscular, skeletal, and connective tissues abnormalities [2].

Distal arthrogryposis (DA) is a clinically heterogeneous subgroup of AMC. It is mostly inherited following an autosomal dominant pattern and is characterized by nonprogressive, congenital contractures involving the distal joints, without any primary neurological or muscular disease. The primary diagnostic criteria are camptodactily, hypoplastic or absent flexion creases, overriding fingers, ulnar deviation of the wrist, talipes equinovarus, calcaneovalgus, and vertical talus.

Distal arthrogryposis type 5D (DA5D) is the only DA type inherited in an autosomal recessive fashion, and it is characterized by severe camptodactily of the hands and wrists, milder camptodactily of the toes, limited knee flexion, talus or varus deformity of the ankles, short stature, scoliosis, ptosis, ophtalmoplegia, astigmatism, micrognathia, tongue groove, and cleft palate [3]. It is due to homozygous or compound heterozygous variants in *ECEL1*. *ECEL1* codifies for the endothelin-converting enzyme-like 1, a transmembrane zinc metalloprotease mostly localized in the endoplasmic reticulum with a slight presentation on the cell surface [4]. Even if little is known about its biological function, it is thought to have a role in the development of the neuromuscular junctions during prenatal life [5,6].

Here, we report a new causative intronic variant detected in two patients presenting with the classical DA5D phenotype (Table 1).

## 2. Results

### 2.1. Clinical Features

Patient 1 is a 13-year-old female, the first daughter of a nonconsanguineous Caucasian couple who were referred to our genetic counseling service for increased risk of age-related aneuploidies in their ongoing pregnancy.

The patient was born after two miscarriages. She was naturally delivered at term with a birth weight of 2910 g (23rd percentile) and a length of 49 cm (47th percentile). During pregnancy, the mother reported decreased fetal movements. Two weeks after birth, the patient was admitted to the neonatal intensive care unit for feeding difficulties that required nasogastric tube feeding. At that time, the physical examination revealed symmetrical knee and elbow arthrogryposis, pterigium colli, and eyelid ptosis, all features that led to the clinical diagnosis of multiple distal arthrogryposis. At ten months of age, she underwent hip surgery for a congenital hip dislocation. Her language and development milestones were reported to be normal except for walking, which was attained at two years of age. At six years of age, the severe arthrogryposis of her knees required a hamstring elongation.

**Figure 1:**
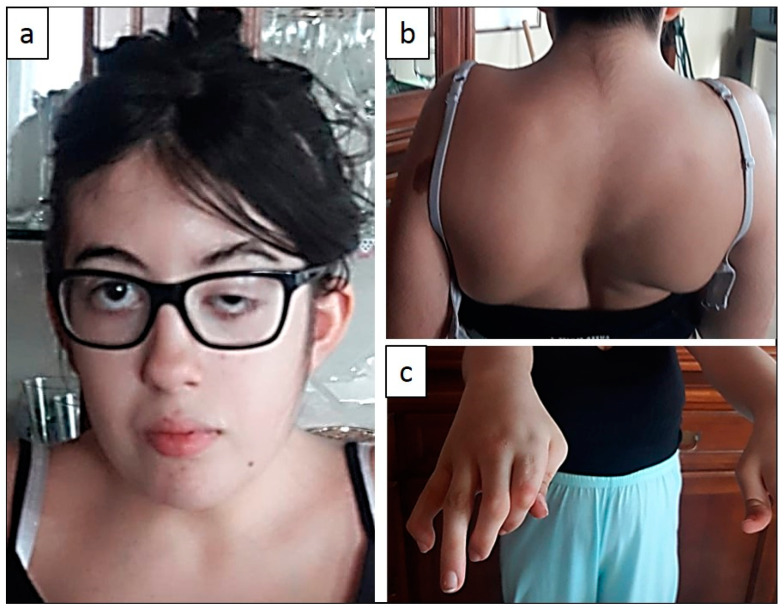
Patient 1. (**a**) Unilateral ptosis, strabismus, and short neck. (**b**) Severe right camptotactyly. (**c**) Left convex scoliosis.

Presently, the patient has mild dysmorphic features (Figure 1) with micrognathia, unilateral ptosis, mouth held open and nasal voice, strabismus, and a short neck. Moreover, she has widespread muscle skeletal abnormalities with contractures limiting the extension of the neck, shoulders, wrists, and knees. She also has axyllae pterygia, scoliosis, hyperlordosis, severe camptodactyly, adducted thumbs, clubfoot, and calcaneovalgus deformity.

**Figure 2:**
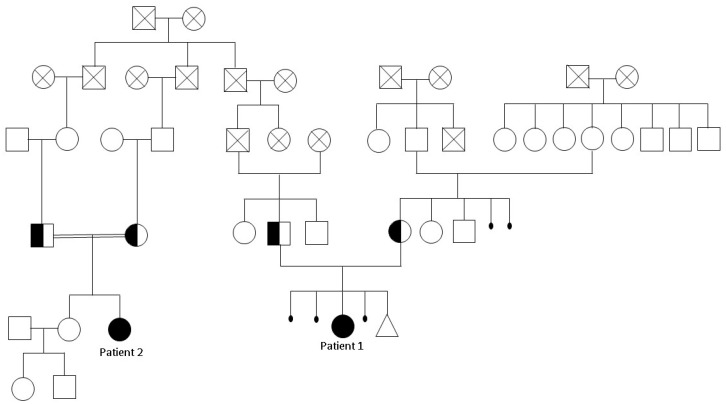
Family pedigree of patients.

Patient 2 is a 39-year-old female, the second daughter of a Caucasian consanguineous couple (i.e., her grandfather and grandmother are first cousins). Furthermore, Patient 2 is distantly related to Patient 1 (whose great-grandfather is the brother of both of Patient 2’s great-grandfathers, Figure 2). Patient 2 was born after a natural delivery with normal growth parameters: 2950 g in weight (26th percentile) and 49 cm in length (47th percentile).

During the pregnancy, decreased and abnormal fetal movements were reported. At ten days, she was admitted to the neonatology unit for the presence of knee, elbow, and ankle contractures that led to the clinical diagnosis of multiple distal arthrogryposis. At the age of one, she had surgery for a correction of bilateral calcaneovalgus clubfoot deformity, and at 18 months, she had surgery for a correction of bilateral hip dislocation. Patient 2’s walking was delayed until the age of two and a half. At five years, she received further surgery for an elongation of the tendons of the fingers.

Patient 2’s language and cognitive development were reported normal. At seven years, scoliosis became evident and was treated conservatively with an orthopedic bust. Recently, she had pneumonia followed by respiratory failure that required tracheostomy.

Presently, the patient has similar but more severe dysmorphic features to Patient 1, with the addition of central tongue atrophy and a furrowed tongue, which is likely responsible for her speech difficulties. Even the pattern of joint and muscle anomalies is similar to Patient 1, with the addition of elbow contractures and decreased muscle build. 

### 2.2. Chromosomal Microarray Analysis and Exome Sequencing

SNP-array analysis was carried out on Patient 1 and Patient 2, who both originated from a village of 5000 inhabitants on the island of Sardinia in Italy and share one common ancestor (Figure 2).

Two different copy number variants (CNVs) were detected: a 153 Kb duplication at 16q23.1 in Patient 1 (arr[GRCh37] 16q23.1(74186894_74340431) ×3) and a 202 triplication at 5q32 in Patient 2 (arr[GRCh37] 5q32(146407693_146610003) ×4). Both CNVs were classified as VoUS (variant of unknown significance) and were not considered responsible for the common phenotype as they were present only in one of the two affected family members. Despite the pedigree of the two families not being suggestive of shared parental inbreeding, the presence of long contiguous stretches of homozygosity (LCSH) in both girls and the common place of origin of the patients’ parents drove the hypothesis of a recessive condition. In particular, a common LCSH was detected in the two patients on the long arm of chromosome 2, at 2q36.3q37.3 (arr[GRCh37] 2q36.3 q37.3(229890930_238851442) ×2 hmz), 9 Mb in size and including 16 OMIM disease causing genes. Among the involved genes, ECEL1 appeared to be a strong candidate because it has already been described in association with distal arthrogryposis (DA5D, OMIM # 615065). Considering the ongoing pregnancy of Patient 1’s mother and the need for a rapid diagnosis, the possible presence of a homozygous sequence variant within ECEL1 was tested by exome sequencing. A biallelic new intronic variant was detected in intron 8–9: NM_004826: c.1507-9G>A. No other known pathogenic variants were detected elsewhere. Sanger sequencing on the parents of both patients revealed the heterozygous presence of the same variant. The analysis of the mutation on the DNA extracted from the amniotic fluid of the mother of Patient 1 was negative. Her pregnancy resulted in the birth of a healthy boy.

### 2.3. ECEL1 Variants

Thirty-eight pathogenic variants have been reported in the ClinVar database so far: 14 of them are missense variants, 6 nonsense, 9 frameshift, 7 splice-affecting, 1 inframe duplication, and 1 inframe deletion. They were detected in 53 patients from 31 families (Table 2).

### 2.4. In Silico Analysis

The new variant detected in our two patients is located in intron 8–9 and is predicted to activate a cryptic splice acceptor site (Human Spicing Finder predictive software, http://www.umd.be/HSF/HSF.html (accessed on 19 February 2021)). The probability of the sequence variant affecting the splicing is also supported by its ADA (adaptive boosting) and RF (random forest) score from dbscSNV, which are 1 and 0.99, respectively [7,8]. When used, the new acceptor site would lead to a 7 bases extension of exon 9, causing a frameshift and introducing a premature termination codon (p.Leu503GlyfsX174) in the luminal domain.

## 3. Discussion

DA5D is the only type of distal arthrogryposis presenting with an autosomic recessive segregation pattern. It is caused by homozygous or compound heterozygous variants in the *ECEL1* gene, which codifies for a member of the neprilysin family of zinc metalloendopeptidases. It is highly expressed in brain and peripheral nerves during early stages of intrauterine development, suggesting that abnormal neuronal development might be involved in the pathogenesis of DA5D. *ECEL1* biallelic deficiency has been shown to affect neuromuscular junctions, resulting in poor contractility [5].

We report about 2 patients presenting with neck, elbow, hand, hip, knee and foot contractures, neck pterygia, camptodactyly, scoliosis and widespread and symmetrical muscle hypotrophy (Table 1).

Genetic analysis showed in both the presence of the intronic homozygous variant c.1507-9G>A (rs797045548) in *ECEL1*. The detected variant has a very low frequency in human population (MAF 0.00004), has not been previously reported in homozygous status and is classified as an uncertain significance variant according to ACMG (American College of Medical Genetics and Genomics and the Association for Molecular Pathology) criteria [9]. The same variant was detected in their parents at the heterozygous status. Three of the four carrier parents shared a common ancestor (Figure 2), while the fourth one was not apparently related with the others. Notably, on GnomAD population databases, no variants in position -9 are reported, while there are several variants in nearby positions (-5, -10), indicating the presence of a conserved sequence.

The *ECEL1* protein product consists of 755 amino acids (UniProt, www.uniprot.org (accessed on 19 February 2021)), with an N-terminal cytoplasmic domain (residues 1–59), a single transmembrane domain (residues 60–82) and a large luminal C-terminal domain (residues 83–775) containing a zinc-binding motif and an active site (residues 612–676).

Thirty gene variants have been described so far in the scientific literature [2,3,10,11,12,13,14,15,16,17,18,19] and a further eight have been reported in ClinVar (https://www.ncbi.nlm.nih.gov/clinvar/ (accessed on 19 February 2021)) as pathogenic (Table 2): 14 missense variants, 9 frameshift, 7 splicing affecting, 6 nonsense, 1 in frame duplication and 1 in frame deletion. No intronic variants have been described so far in DA5D patients. Thirty-five variants out the 38 affect the C-terminal domain, while the remaining 3 are missense variants located within the cytoplasmic N-terminus.

The new variant detected in our two patients is located in intron 8–9. It is predicted to activate a cryptic splice acceptor site leading to a frameshift and a premature stop codon. As this variant maps in C-terminal domain upstream the active site, if expressed, it would yield a truncated protein devoid of its catalytic domain. Alternatively, the presence of a premature stop codon could lead to a non-sense-mediated mRNA decay process. In both these cases, the variant would result in ECEL1 deficiency.

Unfortunately, *ECEL1* is not expressed in blood or in other accessible tissues and the predicted effects of the variant on mRNA could not be verified. However, our patients’ phenotype and the segregation analysis within the families suggests its causative association with the disease. (Figure 2). 

The c.1507-9G>A variant is 5 times more frequent in the panel of 3514 Sardinian sequences (ProgeNIA project) than in human population (MAF 0.0002846 in the Sardinian Pheweb database at https://pheweb.irgb.cnr.it (accessed on 19 February 2021), compared to a MAF 0.00004 in the general population, DbSNP https://www.ncbi.nlm.nih.gov/snp/ (accessed on 19 February 2021)), suggesting that it can be present in our families as a consequence of a founder effect.

Our results expand the genotypic spectrum of mutations in the ECEL1 gene associated with DA5D to include previously unrecognized intronic variants.

## 4. Materials and Methods

The clinical data were obtained in accordance with the ethical standards of Brotzu Hospital (Cagliari, Italy) review board. Informed consent for the case report and the publication of the pictures was obtained.

DNA was extracted from the peripheral blood of Patient 1 and Patient 2 by means of QIAsymphony automatic extractor (QIAGENE, www.qiagen.com (accessed on 19 February 2021)).

Chromosomal Microarray Analysis (CMA) and confirmation tests.

The CMA for Patient 1 and Patient 2 was performed using the Infinium CytoSNP-850K BeadChip (SNP-array, Illumina, San Diego, CA, USA) according to the manufacturer’s protocol. Array scanning data were generated on the Illumina iScan system, and the results were analyzed by Bluefuse Multi 4.4 software.

The confirmation tests on the patients’ DNA were performed by Real-Time PCR on LOC101928035 (Patient 1) and PPP2R2B (Patient 2) using a SYBR Green assay (Livak and Schmittgen, 2001).

Exome sequencing.

Whole exome capture was performed on the patients’ DNA by using the high-throughput NimbleGen SeqCap Exome Enrichment kit (Roche https://www.roche.com/ (accessed on 19 February 2021)) according to the manufacture’s protocol and sequenced on the Illumina NextSeq 550 platform. Sequencing the data alignment to the hg19 human reference genome and variant calling were done with the BWA Genome Alignment Software and GATK Variant Caller (Illumina). Annotating and filtering were performed by Variant Studio software (Illumina, http://variantstudio.software.illumina.com/ (accessed on 19 February 2021)) and Geneyx Analysis software (formerly TGex) (https://pubmed.ncbi.nlm.nih.gov/31888639/ (accessed on 19 February 2021)).

The variants, identified as pathogenic, were confirmed by Sanger sequencing following a standard protocol (BigDye Terminator v3.1 Cycle Sequencing Kit, Applied Biosystems by Life Technologies). The same Sanger sequencing test was extended to the DNA from the peripheral blood of the patients’ parents and the DNA extracted from the amniotic fluid of Patient 1’s mother.

## Figures and Tables

**Table 1:** Clinical Features.

	Clinical Features Reported for DA5D	Patient 1	Patient 2
PRENATAL	Diminished fetal movements	yes	yes
	Intrauterine growth restriction	no	no
	Diminished facial expression	no	yes
	Micrognathia	yes	yes
	Mouth held open	yes	yes
	Ptosis, unilateral or more severe on one side	yes	no
	Strabismus	yes	no
	ophthalmoplegia	no	yes
HEAD AND NECK	Refractive errors	yes	yes
	Central tongue atrophy	no	yes
	Furrowed tongue	no	yes
	Speech difficulties	no	yes
	Nasal voice	yes	yes
	Cleft palate	no	no
	Short neck	yes	yes
	Neck contractures	yes	yes
	Scoliosis	yes	yes
	Hyperlordosis	yes	yes
	Dislocated hips	no	yes
	Contractures of shoulders	yes	yes
	Contractures of elbows	no	yes
SKELETAL AND MUSCLE	Contractures of wrists	yes	yes
	Extension contractures of knees	yes	yes
	Severe camptodactyly	yes	yes
	Adducted thumbs	yes	yes
	Adducted wrists	no	yes
	Clubfoot	yes	yes
	Calcaneovalgus deformity	yes	no
	Decreased muscle mass	no	yes
	Pterygia of neck	yes	yes
SKIN	Pterygia of axillae	yes	yes
	Pterygia of elbows	no	yes
	Pterygia of groin	no	yes
OTHER	Restrictive lung disease	no	yes
	Paroxysmal tachycardia	no	yes

**Table 2:** *ECEL1* variant detected in our patients (green) and variants previously reported as pathogenic (scientific literature and ClinVar database).

	Reference	Variation (NM_004826.4)	Protein Consequence	Status	Type
1	Present study	c.[1507-9G>A]	-	hom	activation of an intronic cryptic acceptor site. potential alteration of splicing
2	Jin et al., (2020)	c.[69C>A];c.[1810G>A]	p.(Cys23Ter);p.(Gly604Arg)	comp het	nonsense + missense
3	Umair et al., (2019)	c.[158C>A]	p.(Pro53Leu)	hom	missense
4	Ullmann et al., (2018)	c.[2005_2006delAC]	p.(Thr669fs)	hom	frameshift
5	Ullmann et al., (2018)	c.[1470G>A]	p.(Trp490Ter)	hom	nonsense
6	Stattin et al., (2018)	c.[1163T>C]	p.(Leu388Pro)	hom	missense
7	Ullmann et al., (2018)	c.[589G>A]	p.(Gly197Ser)	hom	missense
8	Hamzeh et al., (2017), McMillin et al., (2013)	c.[1184G>A]	p.(Arg395Gln)	hom	missense
9	Bayram et al., (2016)	c.[1147C>T]	p.(Gln383Ter)	NA	nonsense
10	Patil et al., (2014), Dohrn et al., (2015), Bayram et al., (2016)	c.[2023G>A]	p.(Ala675Thr)	hom	missense
11	Shaaban et al., (2014)	c.1819G>A	p.(Ser607Gly)	hom	missense
12	Barnett et al., (2014)	c.[1797-1G];[1531G>A]	p.(Gly511Ser)	comp het	splice site + missense
13	Shaheen et al., (2014)	c.1221_1223dup	-	hom	in frame duplication
14	Shaheen et al., (2014)	c.[1210C>T]	p.(Arg404Cys)	hom	missense
15	Shaheen et al., (2014)	c.[1057dupC]	-	hom	frameshift
16	Dieterich et al., (2013)	c.[2278T>C]	p.(Cys760Arg)	hom	missense
17	Dieterich et al., (2013)	c.[1685+1G>T]	p.(Lys552AlafsX33)	hom	splice donor (introducing a premature termination)
18	Dieterich et al., (2013)	c.[1649C>G]	p.(Ser550Ter)	hom	nonsense
19	Dieterich et al., (2013)	c.[1470G>A];[997C>T]	p.(Trp490Ter);(Arg333Ter)	comp het	nonsense + nonsense
20	McMillin et al., (2013)	c.[1252C>T];[590G>A]	p.(Arg418Cys);(Gly197Asp)	comp het	missense + missense
21	McMillin et al., (2013)	c.[1252C>A];[1184+3A>T]	p.(Arg418Ser)	comp het	missense + splice site
22	Dieterich et al., (2013)	c.[966+1G>A]	p.(Asp559AlafsX33)	hom	splice donor (introducing a premature termination)
23	Dieterich et al., (2013)	c.[874delG]	p.[Val292CysfsTer51]	hom	frameshift (premature truncation)
24	McMillin et al., (2013)	c.[869A>G];[797_801delinsGCT]	p.(Try290Cys);(p.Asp266Glyfs15)	comp het	missense + frameshift
25	McMillin et al., (2013)	c.[716dupA]	p.(Tyr239Ter)	hom	frameshift (premature truncation)
26	McMillin et al., (2013)	c.[716dupA];[344_355del]	p.(Tyr239Ter);(Asn115_Ala118del)	comp het	frameshift + in frame deletion
27	reported as path in ClinVar	c.[509del]	p.(Gly170fs)	NA	frameshift
28	reported as path in ClinVar	c.[110_155del]	p.(Phe37fs)	NA	frameshift
29	reported as path in ClinVar	c.[2151+2T>A]	-	NA	splice donor
30	reported as path in ClinVar	c.[1581+1G>A]	-	NA	splice donor
31	reported as path in ClinVar	c.[1506+1G>A]	-	NA	splice donor
32	reported as path in ClinVar	c.[505_529del]	p.(Gly169fs)	NA	frameshift
33	reported as path in ClinVar	c.[278del]	p.(Gly93fs)	NA	frameshift
34	reported as path in ClinVar	c.[4G>T]	p.(Glu2Ter)	NA	nonsense

## Data Availability

The data that support the findings of this study are available on request from the corresponding author. The data are not publicly available due to privacy or ethical restrictions.
